# Modulating the Innate Immune Response to Influenza A Virus: Potential Therapeutic Use of Anti-Inflammatory Drugs

**DOI:** 10.3389/fimmu.2015.00361

**Published:** 2015-07-20

**Authors:** Irene Ramos, Ana Fernandez-Sesma

**Affiliations:** ^1^Department of Microbiology, Icahn School of Medicine at Mount Sinai, New York, NY, USA

**Keywords:** influenza virus, inflammation, innate immunity, ARDS, cytokines, anti-inflammatory therapy

## Abstract

Infection by influenza A viruses (IAV) is frequently characterized by robust inflammation that is usually more pronounced in the case of avian influenza. It is becoming clearer that the morbidity and pathogenesis caused by IAV are consequences of this inflammatory response, with several components of the innate immune system acting as the main players. It has been postulated that using a therapeutic approach to limit the innate immune response in combination with antiviral drugs has the potential to diminish symptoms and tissue damage caused by IAV infection. Indeed, some anti-inflammatory agents have been shown to be effective in animal models in reducing IAV pathology as a proof of principle. The main challenge in developing such therapies is to selectively modulate signaling pathways that contribute to lung injury while maintaining the ability of the host cells to mount an antiviral response to control virus replication. However, the dissection of those pathways is very complex given the numerous components regulated by the same factors (i.e., NF kappa B transcription factors) and the large number of players involved in this regulation, some of which may be undescribed or unknown. This article provides a comprehensive review of the current knowledge regarding the innate immune responses associated with tissue damage by IAV infection, the understanding of which is essential for the development of effective immunomodulatory drugs. Furthermore, we summarize the recent advances on the development and evaluation of such drugs as well as the lessons learned from those studies.

## Introduction

Influenza A virus (IAV) infection usually results in a mild and self-limiting disease that in some individuals, commonly those with underlying medical conditions, can result in complications leading to severe disease and death. Pneumonia, bronchitis, sinus infections, and ear infections are examples of influenza-related complications (1). Thus, influenza has a significant economic impact and is a very important public health concern, with a rate for the 2014–2015 season of 57.1 laboratory-confirmed influenza-associated hospitalizations per 100,000 people reported as of March 14th, 2015 (2). The highest rate of hospitalization is among adults over 65 years old, followed by children under 4 years old, and the average annual influenza-associated deaths in the United States from 1976 to 2007 are 23,607 (3).

Characteristics of the IAV genome, such as its negative-sense, single-stranded segmented RNA, and its airborne transmission in humans provides this virus with a great pandemic potential. The co-circulation of different subtypes in animal reservoirs leads to reassortment (antigenic shift), which may result in a novel subtype that is able to transmit to the human population (4). The circulating IAV subtypes in humans as of 2015 are H1N1 viruses, which caused a pandemic in 2009, and H3N2 viruses; however, several different subtypes have circulated in humans during the last century. The natural hosts of IAV are aquatic birds, which may sporadically transmit viruses to poultry. Humans are, on occasion, infected by these viruses, causing what is known as avian influenza, which is associated with severe disease and high fatality rates (5, 6). The human-to-human transmission in these cases is very limited, and the most important of these IAV are the H5N1 and H7N9 subtypes.

Uncomplicated cases of influenza are limited to attachment and viral replication in the upper respiratory tract, and the symptoms in these cases are nasal obstruction, cough, sore throat, headache, fever, chills, anorexia, and myalgia. These symptoms are the consequences of the inflammation induced upon viral infection (7). Complications of IAV infection are more frequent in people with underlying comorbidities, such as chronic pulmonary or cardiac disease, asthma, immunosuppression, or diabetes mellitus. These complications begin when the viral infection reaches the alveolar epithelium in the lower respiratory tract, where severe tissue damage may occur and affect gas exchange. In alveolar tissue, type I pneumocytes prevent fluid leakage across the alveolar–capillary barrier, and type II pneumocytes resorb fluid from the alveolar lumen and produce lung surfactant (8). Thus, damage of the alveolar epithelium leads to respiratory dysfunction or acute respiratory distress syndrome (ARDS), which often occurs in cases of severe influenza. More extensive discussion on the contribution of the different cell types to tissue damage during influenza infection has been recently published in a very interesting review (9). Most of the lung pathology during influenza virus-induced ARDS is associated with the release of cytokines and other pro-inflammatory mediators, and the contribution of the direct viral cytopathic effect to the alveolar damage is still unclear (10, 11). H5N1 viruses have also been reported to spread to extra-respiratory tissue, although with limited or no viral replication (12).

Influenza complications are also frequently associated with secondary bacterial infections, which may be explained by a series of changes that the virus induces in the lung epithelial cells of the host that predisposes to adherence and invasion as well as changes in the immune response (13–15). For example, it is believed that IAV infection upregulates CD200 receptor in lung myeloid cells, which is involved in negative immune regulation upon interaction with the ligand CD200, resulting in predisposition to secondary bacterial infection (16).

Disease severity caused by IAV infection is greatly associated with high levels of inflammation, with increasing evidence that tissue damage is produced by an exaggerated innate immune response. Thus, many researchers have proposed that treatment with anti-inflammatory therapy could be beneficial. The primary challenge to successfully establish this type of therapy is to downregulate specific mediators of the immune system that have a detrimental effect while avoiding increased levels of viral replication. Here, a review of the innate immunity processes associated with severe cases of IAV infection is provided. Specifically, we discuss clinical studies that have been published regarding the cytokines and chemokines shown to be upregulated in serum or lung tissue of patients with severe disease. We also provide a brief review of the most frequent of those immune mediators, including signaling pathways activated by them and the cellular processes that might lead to tissue damage and disease progression. Finally, anti-inflammatory therapies that have been proposed and tested, either in clinical, preclinical, or *in vitro* studies, are also discussed.

## Innate Immunity to IAV

The first barrier that IAV encounters when invading the host is the mucus layer covering the respiratory and oral epithelia. If the virus successfully overcomes this barrier, it can bind the respiratory epithelial cells, be internalized, and start replicating (17). The cellular defense mechanisms that are initiated upon pathogen invasion involve the sensing of components of pathogens, or pathogen-associated molecular patterns (PAMPs), by pattern-recognition receptors (PRRs) in host cells. This recognition leads to activation of subsequent signaling events that result in the secretion of inflammatory cytokines, type I interferon (IFN), chemokines, and antimicrobial peptides. There are several types of PRRs with a cell-type specific distribution and sub-cellular localizations that may be cytoplasmic, endosomal, or in the plasma membrane.

Cytoplasmic receptors include the retinoic acid-inducible gene I (RIG-I)-like receptors (RLRs), the nucleotide oligomerization domain (NOD)-like receptors (NLRs), and the less-characterized cytosolic DNA sensors (18). These receptors are particularly important in the context of viral infection. Within the RLR family, the most important proteins are RIG-I, melanoma differentiation factor 5 (MDA5), and laboratory of genetics and physiology 2 (LGP2), all of which are expressed in the cytosol of most cell types and participate in the recognition of single-stranded and double-stranded RNA (19). The most studied of the NLRs in the context of virus infection is the NLR family pyrin domain containing 3 (NLRP3), which upon stimulation leads to the activation of the inflammasome system, with important implications in inflammation (20). NLRP3 is expressed in myeloid cell types, such as monocytes, macrophages, dendritic cells (DCs), and neutrophils and in lung epithelial cells (21). Several PAMPs and damage-associated molecular patterns (DAMPs) have been proposed to activate this receptor, including dsRNA (22), the M2 protein of influenza virus (23), and reactive oxygen species (ROS) (24).

Another very important family of PRRs is the toll-like receptors (TLRs). Some of these receptors, such as TLR1, TLR2, TLR4, TLR5, and TLR6, are located in the plasma membrane and are activated mainly by lipids, lipoprotein, and proteins. Other TLRs, namely, TLR3, TLR7, TLR8, and TLR9, are expressed in endosomal compartments and recognize nucleic acids (25). TLRs are highly expressed in antigen-presenting cells, such as DCs and macrophages, and they are also known to be expressed in several T cell subsets (26). For IAV and other RNA viruses, the most important of these TLRs are TLR3 and TLR7/8, which recognize dsRNA and ssRNA, respectively (27).

Other PRRs that are expressed on the cell surface of antigen-presenting cells are the C-type lectin receptors (CLRs), such as the DC-specific intercellular adhesion molecule-3-grabbing non-integrin (DC-SIGN) or dectin-1 and dectin-2. CLRs recognize carbohydrate ligands and are also mainly expressed in antigen-presenting cells (28). Several reports have shown an interaction between IAV and DC-SIGN (29–31), which would facilitate infection of DCs.

Recognition of PAMPs by these PRRs leads to the activation of multiple signaling cascades initiating the innate immune response. This response leads to the production of type I and type III IFNs. Binding of these IFNs to their receptors in a paracrine or autocrine manner leads to the establishment of an antiviral response, characterized by the expression of hundreds of genes that will counteract viral replication (32). Also, PAMP sensing results in the release of pro-inflammatory cytokines and chemokines by the cells that will contribute to the development and modulation of specific T cell responses and recruitment of different immune cells, such as monocytes, neutrophils, and natural killer (NK) cells, to the infected tissue. In the case of antigen-presenting cells, such as DCs and some subtypes of macrophages, they also undergo maturation and migrate to the secondary lymphoid organs where antigen is presented to T (33) and B cells (34). These adaptive immune responses initiated upon innate immune activation are known to be necessary for protection and viral clearance, as recently reviewed by Chiu and Openshaw (35).

Hence, in the current model of IAV-induced ARDS, IAV particles invade a new host and if, able to cross the mucosal barrier, will infect tissue cells in the upper respiratory tract. In some cases, the virus reaches the lung, where it can infect type I and II pneumocytes, endothelial cells, and immune cells (9, 36–38). The presence of the virus is detected by infected cells, which release cytokines, chemokines, and other mediators in order to control the infection and remove dead cells and stimulate the initiation of adaptive immune responses. However, other effects of those mediators, which are described in detail below, are detrimental for the integrity of the tissue (11, 39).

While these alert systems are aimed to mount an effective immune response to clear viral infection, there are also important negative consequences of those responses that might compromise tissue integrity. One of the most described of those consequences is the production of ROS. Pro-inflammatory mediators induce intracellular ROS by activating the nicotinamide adenine dinucleotide phosphate (NADPH) oxidase activity. Also, work by Ye et al. has shown that inhibiting ROS production *in vitro* results in attenuation of release of pro-inflammatory cytokines (40, 41), thereby amplifying the immune response. In addition to reacting with DNA, proteins, and lipids resulting in structural cell and tissue damage, ROS are known to be the second messengers that participate in several signaling pathways and function as transcriptional regulators (42). It is also known that pro-inflammatory responses activate signaling pathways that result in the activation of apoptosis and necrosis (43, 44). Accordingly, apoptotic alveolar epithelial cells have been observed by histochemistry of lung tissue from two patients who died by H5N1 infection (12).

## Hypercytokinemia and Pathogenesis in Severe Cases of Human Influenza

Several studies have characterized the profile of cytokines in human cases of influenza in order to understand the connection between innate immunity and pathogenesis. In cases of seasonal influenza, complications are mostly associated with secondary bacterial infection. Most cases of severe primary viral pneumonia have been associated with pandemic influenza, such as 2009 H1N1 or 1918 H1N1 influenza virus, and cases of avian influenza, such as infections by H5N1 or H7N9 influenza viruses (45, 46).

Acute respiratory distress syndrome is the main cause of death in IAV-infected patients (47, 48). Histopathology caused by complicated IAV infection in the absence of bacterial pneumonia consists of inflammation, congestion, epithelial necrosis of the larger airways, and diffuse alveolar damage characterized by hyaline membranes, interstitial and intra-alveolar edema, necrotizing bronchitis and bronchiolitis, and in some cases, hemorrhage (49, 50). Autopsies from fatal cases of 1918 H1N1, H5N1, and the 2009 H1N1 pandemic virus show comparable pathological characteristics (47, 50). Fatal infection with H7N9 influenza viruses in humans also showed diffuse alveolar damage as one of the main histopathology findings (51).

The majority of the patients infected by pandemic 2009 H1N1 virus experienced a mild disease with influenza-like symptoms that typically resolved in a few weeks (47, 48). However, due to the lack of pre-existing immunity against this virus, complications of the disease occurred in some patients, mostly those with underlying conditions (47). Gao et al. found the levels of seven proteins markedly upregulated in lung tissue in fatal cases of influenza virus 2009 H1N1 infection. Those proteins are interleukin (IL)1 receptor antagonist protein (IL1RA), IL6, tumor necrosis factor (TNF)-α, IL8, monocyte chemoattractant protein 1 (MCP1), macrophage inflammatory protein (MIP) 1β, and IFNγ-inducible protein-10 (IP10)(52). In this work, they also found high levels of apoptosis in the lungs and airway by terminal deoxynucleotidyl transferase dUTP nick end labeling (TUNEL) staining, as well as marked levels of cleaved caspase 3 (52). A similar study by To et al. showed significantly higher levels of granulocyte colony-stimulating factor (G-CSF), IFNα2, IL1α, IL6, IL8, IL10, IL15, IP10, and MCP1 in plasma samples of patients that developed ARDS and died than in those patients that developed mild disease at early times after onset of symptoms (48). High levels of IP10, MCP1, and MIP1β were also found in a separate group of patients infected by 2009 H1N1 influenza virus (53). In this study, elevated levels of IL8, IL9, IL17, IL6, TNFα, IL15, and IL12p70 were found specifically in patients that required hospitalization, and IL6, IL15, and IL12 were markers of severe disease. In agreement, other studies reported high levels of IL6, IL8, IL10, and the chemokine MCP1 in 2009 H1N1 virus-infected patients (54) and correlated serum levels of IL6 and IL1β with disease severity in children infected by the same virus (55). An additional report showed elevated levels of IL2, IL12, IL6, IL10, IL17, and IL23 in patients with severe disease and correlation between clinical manifestations and IL6 and IL10 serum levels in patients infected by 2009 H1N1 IAV (56). Other studies reporting similar results are summarized in Table 1 (54, 57, 58).

**Table 1:** **Cytokines and chemokines detected in serum or lung tissue samples of human subjects with severe disease infected by IAV**.

IAV subtype	Cytokines	Chemokines	Reference
2009 H1N1	IL6, TNFα, IL9, IL17, IL15, and IL12	IL8	(53)
2009 H1N1	IFNα2, IL1α, IL6, IL10, and IL15	IL8, IP10, and MCP1	(48)
2009 H1N1	IL6, TNFα, and IL15	IL8	(57)
2009 H1N1	IL2, IL12, IL6, IL10, IL17, and IL23		(56)
2009 H1N1	IL6 and TNFα	IL8, IP10, MCP1, and MIP1β	(52)
2009 H1N1	IL6 and IL1b		(55)
2009 H1N1	IL6 and IL10	IL8 and MCP1	(54)
H3N2	IL6, TNFα, and IL33		(58)
H5N1		IP10 and MIG	(59)
H5N1	IL6, IL10, IFNγ	IL8, IP10, MCP1, and MIG	(60)
H5N1	IFNβ, IL6, IFNγ, and TNFα	IL8, IP10, MCP1, RANTES, MIP1α, and MIG	(61)
H7N9	IL6	IL8 and MIP1β	(62)
H7N9	IL6	IL8 and MIP1β	(63)

Cytokine responses in H5N1-infected patients have also been studied. Peiris et al. found elevated levels of IP10 and monokine induced by IFNγ (MIG) in serum of H5N1-infected patients (59). Similarly, de Jong et al. found the levels of IP-10, MIG, and MCP1 elevated in patients with H5N1 infections (60). Interestingly, in both studies, they found large numbers of macrophages infiltrated in the lung, in accordance with the functions of those chemokines. de Jong et al. also found elevated levels of IL6, IL8, IL10, and IFNγ in those patients (60). The level of cytokines was associated with elevated levels of viral replication. Another study that evaluated the levels of cytokines in two fatal cases of H5N1 infection found high levels of regulated on activation, normal T cell expressed and secreted (RANTES), MCP1, MIP1α, IP10, IL8, MIG, IFNβ, IL6, IFNγ, and TNFα in the lungs and serum in one patient, while no cytokine expression was detected in the other case (61). However, the patient who did not show cytokine expression was pregnant and treatment with glucocorticoids was provided in both cases, which may have affected the immune response although it is unclear how these or other factors could have affected the results (61).

Information regarding H7N9 IAV infections in humans is more limited given the recentness of the outbreak. However, a study evaluating the cytokine responses in infected patients identified early high levels of IL6, IL8, and MIP1β in serum as predictive parameters of severe or fatal outcome (62). Another study found a positive correlation of the same molecules (IL6, IL8, and MIP1β) with pharyngeal virus load in H7N9-infected patients (63).

Most of these studies with human samples point to elevated levels of cytokines and chemokines in IAV-infected patients. Interestingly, there is a clear overlap in the cytokines that are observed in most of those studies. A summary of the cytokines and chemokines found to be upregulated in humans infected by IAV is provided in Table 1. Experiments performed *in vitro* also have identified the production of similar cytokines in different systems, including IL6, TNFα, IFNs, IL1β, RANTES, IL8, MIP1β, and MCP1 (64, 65). Since the reported data indicate that the induction of these molecules might be associated with pathogenesis, understanding the effects of those proteins in receptor-expressing cells and the signaling pathways that they induce is important for eventually translating that information to the identification of efficient and safe treatment alternatives. Therefore, in the next section, we focus on the functions of each one of those cytokines and chemokines in more detail, as well as their participation in tissue damage in other diseases or other models as an additional indicator of their pathogenic potential.

## Cytokines and Chemokines with Increased Expression during Severe Influenza: How They Work and Their Involvement in Tissue Damage

Upon influenza infection, viral PAMPs are sensed by the cells and multiple signaling pathways are activated as a part of the innate immune response. The purpose of the innate immune response is to lead to the clearance of viruses and infected cells, as well as the activation of the adaptive immune response. However, these events can also result in tissue destruction as a consequence of excessive activation. Data discussed in the previous section indicate an association between the activation of the innate immune response, typically measured as the production of cytokines and chemokines in serum, and a more severe pathogenesis or fatality in many cases, supporting the hypothesis of causative relationship between innate immunity and severe disease. To provide deeper insights into these events and their connection, in this section we will review the effects and signaling pathways associated with the production of the main cytokines upregulated during influenza infection. Because of the broad and numerous functions of these cytokines, it is a challenging task to parse their functions as many of them are redundant and regulated by complex networks involving multiple transcription factors, adaptors, or secondary mediators. In terms of their potential as therapeutic targets, some therapies using monoclonal antibodies to neutralize the damaging effects of those proteins have been developed and are already in the clinic for treatment of anti-inflammatory diseases, while other approaches, such as administration of pro-inflammatory cytokines, small molecules, siRNA or shRNA, or gene therapy, are under study (66).

### Cytokines

#### TNFα

TNFα is the most studied of the cytokines, since it is involved in a large number of functions with multiple effects, such as activation of inflammatory responses, stimulation of adaptive immunity, cell survival, apoptosis, proliferation, and cell differentiation (67, 68).

The receptors for TNFα are TNF-R1, which is constitutively expressed in most cell types, and TNF-R2, which is expressed in immune cells (67). Binding of TNFα to its receptor results in the activation of multiple intracellular signaling pathways, which have been extensively reviewed elsewhere (68, 69). Therefore, in this review, we provide a general overview of these processes and the related outcomes in terms of tissue damage and pathogenesis.

TNFα leads to the activation of nuclear factor kappa-light-chain-enhancer of activated B cells (NF-κB) pathway, which promotes the expression of a large number of inflammatory genes. In the classical NF-κB pathway, which is the one activated upon TNFα engagement, NF-κβ is a dimer made up of two subunits, p50 and p65. In a resting state, NF-κB is inactive in the cytoplasm, forming a complex with the inhibitor of nuclear factor κB (IκB). Stimulation that activates the pathway results in the degradation of IκB, allowing the p50/p65 dimer to translocate to the nucleus where it interacts with DNA, leading to the regulation of gene expression (70). Other stimuli that activate NF-κB transcription factors are viral genomic RNA or DNA, bacterial products, acidic pH, and stress-related molecules, such as ROS among others. In addition, it is known that endogenous or host-derived ligands that are generated during tissue damage are also sensed by cell surface receptors, leading to NF-κB activation (71).

Engagement of TNFα to its receptor also leads to the induction of apoptosis. This occurs by several mechanisms, but the major one involves recruitment of pro-caspase 8 to TNF-R1 through the adaptors fas-associated death domain (FADD) and the TNF-R superfamily member 1A (TNFRSF1A)-associated death domain (TRADD), which leads to the auto-cleavage of caspase 8 to its active form. These events then result in caspase 3 activation and induction of apoptosis. Caspase 8 also leads to the release of cytochrome *c* from the mitochondria, contributing to apoptosis induction through the “intrinsic pathway” (68, 72). Interestingly, TNFα also induces caspase-independent cell death by a mechanism involving receptor-interacting serine/threonine-protein kinase 1 (RIPK1) by a kinase-regulated process, and it is known as necroptosis (73–75).

Another described function for TNFα is to stimulate the production of ROS (73, 74, 76), which are also inducers of apoptosis and necrosis (76). In addition, TNF signaling stimulates the activity of the NADPH oxidases (Nox) in neutrophils and macrophages, such as NOX2, resulting in the generation of superoxide $\left({\text{O}}_{2}^{-}\right)$ (77, 78), which is important for clearing intracellular microorganisms (74, 79).

On the other hand, TNFα signaling leads to activation of the c-jun NH2-terminal kinase (JNK) that also regulates several cellular functions including apoptosis, survival, and cell growth by phosphorylating downstream transcription factors, such as c-jun, activating transcription factor 2 (ATF2), or nuclear factor of activated T cells (NFAT). Interestingly, ROS has also been shown to be a co-activator of TNF-induced JNK activation and cell death (76).

Given the multiple functions of TNFα in inflammation and tissue damage, it is a very important target for immunomodulatory therapy in general. Indeed, antibodies that block its function are used as a primary treatment in some autoimmune disorders, such as rheumatoid arthritis (RA) and Crohn’s disease, and several blocking agents are already approved and used in the clinic for such disorders (80). In the case of influenza disease, TNFα-blocking agents have also been tested for treatment of IAV-induced inflammation. Mice treated with one of these agents, etanercept, showed reduced lung inflammation and morbidity after challenge with influenza virus (81).

#### IL6

IL6 has been attributed to both pro-inflammatory and anti-inflammatory effects (82, 83). In addition, IL6 is involved in the regulation of metabolism, bone homeostasis, and neural processes. The production of IL6 is tightly regulated, and its continuous production has been associated with numerous chronic and autoimmune diseases. The synthesis of this cytokine is upregulated during infection or stress, and its major roles involve the production of acute phase proteins by hepatocytes and stimulation of the adaptive response by inducing the differentiation of activated B cells and CD4^+^ T cells (84).

Activation of IL6 signaling may take place through classic or trans-signaling pathways. In the classical activation, IL6 interacts with membrane-bound IL6 α-receptor (IL6R), while in the trans-activation pathway, IL6R is soluble. In both scenarios, the signal-transducing β-subunit glycoprotein gp130 forms part of the receptor complex and plays a fundamental role in initiating the signal. IL6R is expressed in a limited number of cells types, namely macrophages, neutrophils, some types of T-cells, and hepatocytes (85). However, gp130 is ubiquitously expressed, allowing IL6 signaling to take place in a broad range of tissues. It is believed that trans-signaling accounts for the pro-inflammatory effects of IL6, while the classic signaling is more associated with anti-inflammatory effects. Therefore, this dual activity has interesting implications when considering IL6/IL6R as a therapeutic target. A very interesting review by Scheller et al. discusses the dual functionality of classic versus trans-signaling (83).

Dimerization of gp130 leads to Janus kinases (JAK) activation, which results in phosphorylation of tyrosine residues in the cytoplasmic region of gp130. Next, the signal transducer and activator of transcription (STAT) 3 is phosphorylated, dimerizes, and translocates to the nucleus to regulate the expression of multiple genes associated with the induction of cell growth, differentiation, and survival (86). On the other hand, phosphatase Src homology domains containing tyrosine phosphatase (SHP)2 are recruited, leading to activation of the mitogen-activated protein kinase (MAPK) pathway, including ERK1/2 (associated with survival), p38, and JNK (associated with stress). IL6 can also lead to the activation of phosphatidylinositol-4,5-bisphosphate 3-kinase (PI3K) (87), which is classically associated with survival and cell growth. An important group of genes that are also regulated by JAK/STAT3 IL6-mediated activation is the suppressor of cytokine signaling (SOCS) family. Specifically, SOCS1 and 3, which are the most related with IL6 activation, inhibit the phosphorylation of gp130, resulting in a blockade of the JAK/STAT pathway (86).

Primarily, IL6 (but also IL1α/β and TNFα) is a potent inducer of the synthesis and release of approximately 30 proteins known as the acute phase proteins (88, 89). Acute phase proteins are secreted mainly by hepatocytes, and have multiple immunomodulatory effects. These proteins include the C-reactive protein (CRP), serum amyloid P component (SAP), mannose-binding protein, α1 antitrypsin, α1 antichymotrypsin, α2 macroglobulin, fibrinogen, prothrombin, and complement factors, among others. They are structurally and chemically unrelated, and there is a broad amplitude in their physiological functions, which ranges from inhibition of pathogen growth, facilitation of their removal by phagocytic cells, and elimination of infected cells to other unrelated functions, such as providing anti-inflammatory feedback to the immune system or modulation of coagulation (90). CRP is perhaps the most studied of these proteins, and it is frequently used as a diagnostic marker for inflammation. Interestingly, CRP is known to be released locally by cells of the respiratory epithelia and the liver in response to cytokine stimulation and that patients with ARDS have high levels of CRP (91). CRP was identified as a biomarker of disease severity in patients hospitalized with IAV infection at the time of admission (92). However, another study indicates that, although the levels of CRP are elevated in patients with acute lung injury, a higher level of plasma CRP predicts a more favorable outcome in adult patients (93). This protein has both pro-inflammatory and anti-inflammatory functions, and its function remains to be well characterized. Chronic overexpression of these acute phase proteins is also characteristic of some chronic, autoimmune pro-inflammatory diseases, such as RA.

Excessive production of IL6 has been associated with several pathological manifestations, such as Castleman disease or RA. For this reason, IL6 has been extensively investigated as a therapeutic target, leading to the development of monoclonal antibodies, such as tocilizumab, which has already been approved for the treatment of these diseases (94). However, in the case of IAV infection, IL6 seems to have a protective role in the mouse model, promoting viral clearance and limiting inflammation (95), indicating that IL6 blocking agents might not be adequate for inflammation treatment during IAV infection.

#### IL1β

IL1β belongs to the broad IL1 family. The precursor of IL1β (pro-ILβ) is processed by caspase 1, which activation is mediated by the action of the inflammasome. IL1β is produced by immune cells, such as monocytes, tissue macrophages, and skin DCs in response to TLR activation, complement components, and other cytokines, such as TNFα (96).

The receptor for IL1β, as well as for IL1α, is IL1 receptor type I (IL1RI). The IL1RI presents a toll-IL1-receptor domain (TIR), which is also present in TLRs, and it is necessary for signal transduction. Engagement of IL1β/α to IL1RI leads to the recruitment of the co-receptor chain IL1R accessory protein IL1RAcP. Next, the adaptor protein myeloid differentiation primary response gene 88 (MyD88) interacts with the TIR domain, leading to phosphorylation of the IL1RI-associated kinases, IRAKs. Further phosphorylation steps involving the inhibitor of NF-κB kinase α and β (IKKα/β) and the NF-kappa-B essential modulator (NEMO) lead to the subsequent activation of the NF-κB transcription factors (97). JNK and p38 MAPK pathways are also activated upon IL1RI engagement (98). These events result in the induction of the expression of inflammatory genes including IL1α and β, as well as IL6 and RANTES among others, leading to an amplification loop.

While the main function of this cytokine is to mediate inflammation through activation of the NF-κB transcription factors, IL1β signaling has other additional consequences. For instance, the activation of IL1RI include increased expression of cyclooxygenase-2 (COX-2), inducible nitric oxide synthases (iNOS), prostaglandin 2 (PEG2) (71), and adhesion molecules, such as intercellular adhesion molecule-1 (ICAM-1) on mesenchymal cells and vascular-cell adhesion molecule-1 (VCAM-1) on endothelial cells. This latter property promotes the infiltration of inflammatory cells into the extravascular space (99).

IL1 cytokines are highly associated with acute and chronic inflammatory afflictions. As such, therapies to counteract the effect of this cytokine have been developed and are under study. In particular, treatment with an IL1R antagonist (IL1Ra), known under the generic name anakinra, has been approved to relieve symptoms and pain in patients with RA, and it is a standard therapy for autoimmune syndromes in general (96, 100).

Several studies suggest that IL1β has important roles in tissue damage in several mouse models of inflammation, including induction of systemic inflammation with turpentine or zymosan-induced peritonitis (101). There is also data indicating an excessive activation of the inflammasome in lung pathology – which is activated by the PB1-F2 protein of influenza virus (102), probably as a consequence of subsequent NF-κB activation. Interestingly, another study showed a positive effect on survival after administrating the IL1Ra to influenza virus-infected mice (103). However, in another model of influenza virus infection, while IL1β-infected mice showed reduced body temperature, mortality was higher in IL1β knock out mice (104). In agreement with this, more recent studies have suggested that the inflammasome, in which IL1β has an important role, is important for mediating healing and reducing lung damage, while it is not necessary for virus clearance or humoral adaptive immune responses (105). Indeed, there is increasing evidence that IL1β has an important role in epithelial repair in patients with ARDS (106–108) and this effect seems to be mediated by epidermal growth factor (EGF)/transforming growth factor-α (TGF-α) pathway (109). More recently, it has been shown that IL1β activates the expression of the early growth response (Egr)1 transcription factor through activation of the EGF receptor (EGFR) (110).

Therefore, excessive IL1β responses might contribute to lung injury during severe cases of influenza, but its role in tissue repair seems to be necessary to ensure recovery. Therapeutic strategies targeting this aspect of pathogenesis are complicated given the dual role of IL1β signaling in inflammation and in tissue repair, and a better understanding of the mechanisms of action of IL1β and consequences of altering its functions is needed.

#### Type I and Type III IFN

The most important function of type I and type III IFN is to induce the activation of an antiviral state in infected and neighboring cells. For this reason, these cytokines are very important for protecting against acute viral infections. In addition, type I IFNs have also an important role in the stimulation of adaptive immunity (111, 112).

The most studied type I IFNs are IFNβ, expressed by virtually all cells, and IFNα, produced primarily by hematopoietic cells. Both IFNα and β interact with the IFNα/β receptor (IFNAR), which results in the activation of the receptor-associated protein tyrosine kinases (JAK1) and tyrosine kinase 2 (TYK2). Then, the transcription factors, STAT1 and STAT2, are phosphorylated, dimerize, and translocate to the nucleus (113), where they assemble with IFN-regulatory factor 9 (IRF9) to form the complex IFN-stimulated gene factor 3 (ISGF3). This complex binds specific sequences in the DNA and promotes transcription of hundreds of ISGs, which leads to numerous changes in the transcriptome of the cell thus activating the antiviral response (114, 115). Also, under certain conditions, type I IFNs are able to induce the formation and phosphorylation of STAT1 homodimers, which may bind gamma-activated sequences (GAS) and induce the expression of a different set of genes (116). This GAS-stimulated gene response is mainly activated by type II IFN as described below, and is composed principally of pro-inflammatory genes. Interestingly, type I IFN signaling also leads to STAT3 phosphorylation, which downregulates type I IFN-mediated induction of inflammatory mediators (such as MIG and IP10) while supporting ISGF3-dependent induction of antiviral genes (117).

Type III IFN or IFNλ is a more recently discovered antiviral IFN that triggers STAT1 activation through engagement of an independent heterodimeric receptor, IL-28 receptor α/IL-10 receptor β (IL28Rα/IL10Rβ) complex (118, 119), found primarily on epithelial cells of both the respiratory and gastrointestinal tract. There are three IFNλ proteins, IFNλ-1, -2, and -3 (also known as IL29, IL28A, and IL28B, respectively), all of which signal through the same receptor. Signaling through type III IFN receptor complex results in a cascade of signals similar to that produced by ligation of the type I IFN receptor, which are mediated by JAK1 and TYK2, leading to the formation of a transcription factor complex, ISGF3. Therefore, the biological responses induced by type I and type III IFNs are very similar and mainly characterized by the induction of antiviral responses with the main difference between them being the expression of the receptor in different cell types (120).

While type I IFN is known to be a key mediator of virus clearance during influenza virus infection (121), excessive IFN signaling has detrimental effects on disease severity, mostly as a result of overall increased inflammation (pro-inflammatory cytokines and lung-infiltrating cells), cell death, and oxidative stress that might have damaging effects on the host (122, 123). The production of type I IFN and its pathological effects are supported by its role in other immune diseases. In particular, genetic and transcriptomic analysis of blood from systemic lupus erythematosus (SLE) patients, has attributed type I IFN a central role in the pathogenesis of this disease (124). Type I IFN has also been implicated in the pathogenesis of RA (125) and a type IFN I signature has been documented in patients with Aicardi–Goutieres syndrome (126). It has been shown that the type I IFN receptor sensitizes macrophages to death caused by *L. monocytogenes* infection (127). Interestingly, type I IFN also has been associated with endothelial dysfunction through induction of endothelial nitric oxide synthase (128). However, the mechanisms of type I IFN-mediated regulation of oxidative stress have not been analyzed in detail.

While the damaging potential of type I IFN is evident, the main feature of this family of cytokines is that they are crucial inducers of the antiviral response and therefore they are absolutely required to fight IAV infection. Studies performed in mice clearly indicate that viral replication and disease severity are increased in the absence of IFN, indicating that both type I and type III IFN having protective roles (129). Given the importance of type I IFN induction in defeating viral infection at the cellular level, desirable anti-inflammatory therapies to treat IAV or other viral infections should not fully blunt this type of response.

#### Type II IFN

Interferon-γ is the only member of the type II IFN family and is mainly produced by T cells and NK cells. The production of IFNγ is controlled by IL12 and IL18 released by antigen-presenting cells, such as DCs and macrophages. Type II IFN plays important roles in the stimulation of antigen presentation by macrophages, in activating the cellular Th1 responses upon infection by intracellular pathogens, and in regulating B cell functions (130).

The IFNγ receptor (IFNGR) comprises two different subunits, IFNGR1 and IFNGR2. Activation results in signal transduction through JAK1 and JAK2 and subsequent phosphorylation and homo-dimerization of STAT1 transcription factors. STAT1 dimers subsequently translocate to the nucleus and activate the GAS elements (131), which lead to the expression of IFNγ-related genes. Interestingly, some of these genes are transcription factors (such as IRF1) that can lead to the activation of a second wave of genes (such as IFNβ) (130) and thus there is significant overlap between the IFNα/β- and IFNγ-regulated genes.

One of the most important functions of IFNγ is that it stimulates antigen presentation by several mechanisms. Thus, IFNγ upregulates the expression of the major histocompatibility complex (MHC) class I (132) and MHC class II (133). Interestingly, IFNγ also facilitates antigen processing by stimulating the expression of several molecules associated with this function, such as proteasome subunits, including LMP2 and LMP7 (134, 135) or of the regulator of the immunoproteosome proteasome activator (PA) 28 (136). At the cellular level, activation of IFNγ signaling promotes cell growth and proliferation, but also it has been shown to be important in the upregulation of pro-apoptotic molecules [such as protein kinase R (PKR), the death associated proteins (DAPs), cathepsin D, and surface expression of the TNFα receptor]. A very important consequence of the activation of macrophages and neutrophils by IFN is the enhancement of microbial killing processes, mainly mediated by induction of the NADPH-dependent phagocyte oxidase system or respiratory burst (release of ROS), stimulation of NO production, and upregulation of lysosomal enzymes (137, 138). This defense mechanism, however, is also damaging for infected tissues and has been shown to enhance the pathogenesis during IAV infections (41, 139). IFNγ has also implicated in the pathology of diseases, such as systemic lupus erythematous (140) or multiple sclerosis (141).

### Chemokines

Chemokines are small chemotactic cytokines that play important roles in driving many components of inflammation, the most important of which is leukocyte migration. Chemokine receptors in the cell surface are transmembrane G protein-coupled receptors (GPCRs), and their activation leads to the transduction of intracellular signaling pathways that promote migration toward the chemokine source. Other functions mediated by chemokines include regulation of cell viability, proliferation, differentiation, and migration (142). The chemokine system is very promiscuous in providing flexibility and specificity in the trafficking of immune cells, and a specific chemokine may act on several leukocyte populations to coordinate the recruitment of cells with related functions.

#### RANTES

RANTES also known as chemokine (C–C motif) ligand 5 (CCL5), plays an active role in recruiting leukocytes to inflammatory sites. In particular, it has been shown to induce the migration and recruitment of T cells, DCs, macrophages, monocytes, eosinophils, NK cells, mast cells, and basophils (143–146). Also, it induces the proliferation and activation of certain NK cells. RANTES is produced by macrophages, DCs, T lymphocytes, platelets, eosinophils, fibroblasts, endothelial, and epithelial cells. In general, production of RANTES is associated with viral infections. Interestingly, RANTES is a co-receptor for HIV (147) and for this reason, there is a field of intensive research to develop pharmacological inhibitors of this receptor with the ultimate goal of producing a therapeutic agent (148, 149). High levels of RANTES have also been associated with extensive inflammation of the lung in cases of avian influenza (150) and other viral infections. Deficiency of the receptor for this chemokine, CCR5, which is also the receptor for MIP1α and MIP1β, resulted in increased mortality in IAV-infected mice, suggesting that the function of those chemokines is important for virus clearance, and therefore, they are not promising targets to reduce inflammation (151).

#### IP10

IP10 or (C–X–C motif) ligand (CXCL) 10 is a protein highly associated with the presence of viral infection. Several cell types release IP10, including T lymphocytes, neutrophils, monocytes, DCs, endothelial and epithelial cells, and fibroblasts. IP10 expression is induced by IFN-γ and the gene features ISRE and NF-κB binding sites in the promoter (152), allowing for direct upregulation upon virus infection (153). IP10 interacts with the C–X–C receptor (CXCR) 3 to activate the main target cells, which include T and B lymphocytes, NK cells, DCs, and macrophages. As a consequence of this interaction, signal transduction leads to chemotaxis toward inflamed or infected areas, apoptosis, and proliferation or cell growth inhibition (154). IP10 is known to contribute to the pathogenesis of several infectious diseases (154) and of many autoimmune diseases, such as type 1 diabetes, RA, psoriatic arthritis, or SLE (155). Experiments in mice have shown that the lack of IP10 or its receptor reduces the severity of ARDS during influenza virus infection, suggesting the potential of this signaling pathway as a therapeutic target for ARDS treatment (156).

#### IL8

IL8 or CXCL8 is a potent neutrophil attractant and activator, but also acts on monocytes and mast cells, which express the IL8 receptors, CXCR1 and CXCR2. This chemokine is mainly produced by macrophages, epithelial cells, and endothelial cells (157). Interestingly, monocytes and macrophages produce low amounts of IL8 during influenza virus infection (158), while epithelial cells produce high levels of IL8 *in vitro* (159). Several transcription factors activated upon viral recognition have been shown to bind IL8 promoter and stimulate IL8 production. These include NF-κB, the activator protein 1 (AP-1), the CCAAT-enhancer-binding protein (C/EBP)-β, IRF1, and IRF3 (160, 161). IL8 has a significant role in ARDS, which is characterized by a large influx of neutrophils to the lung during severe influenza (162). Neutrophils protect against microbial infection through the release numerous factors such as ROS, proteinases, and neutrophil extracellular traps, molecules that, when produced in excess, might also have damaging effects (163). In addition to the contribution of IL8 to pathogenesis through increased inflammation via neutrophil recruitment, patients with ARDS also have been shown to present auto-IL8 antibodies that complex with IL8. These complexes are also able to induce chemoattraction of neutrophils, but interestingly, they trigger superoxide and myeloperoxidase release (neutrophil respiratory burst and degranulation) from human neutrophils in a FcγRIIa-dependent way (164).

#### MCP1

MCP1 or CCL2 regulates the migration and infiltration of cells expressing the receptor CCR2, which includes monocytes, memory T lymphocytes, and NK cells, and is produced either constitutively or after induction by oxidative stress or pro-inflammatory mediators. It also participates in the phenotypic polarization of memory T cells toward a Th2 phenotype (165, 166). MCP1 is produced by several different cell types, including endothelial, fibroblast, epithelial, smooth muscle, and monocyte cells among others, monocyte and macrophages being the main sources (167). This chemokine has been implicated in the pathogenesis of several diseases, such as asthma (168), RA, cardiovascular diseases, cancer (169), and some neuropathologies (170). CCR2 signaling results in the activation of PI3K, MAPK, and protein kinase C, and therefore, elicits a broad range of cellular responses (171, 172). In the context of IAV infection, conflicting results have been reported regarding MCP1 function. On the one hand, one study showed that neutralization of MCP1 *in vivo* reduced the immunopathology in a mouse model (173). However, a different report showed increased alveolar epithelial damage and apoptosis upon a similar treatment (174). A separate report showed that CCR2^−/−^ mice infected with IAV presented decreased pathological signs, but higher pulmonary titers early after infection (151). Thus, further characterization on the role of this chemokine is necessary to determine its potential as a target for anti-inflammatory therapy.

#### MIP1β

Macrophage inflammatory protein-1β or CCL4 is also involved in the recruitment of multiple immune cells, such as monocytes, T-lymphocytes, monocytes, eosinophils, basophils, DCs, and NK cells (175). It also induces activation of these cells and increased adhesion (176). Low levels are constitutively expressed but its production is activated by multiple inducers (such as PAMPs and cytokines) in different cell types, including monocytes, macrophages, neutrophils, DCs, epithelial cells, fibroblast, and multiple cells from the nervous system (175). The receptor for MIP1β is CCR5, although it is known that a natural truncated form of MIP1β, which lacks two N-terminal amino acids, also binds and signals through CCR1 and CCR2B (177). Associations with autoimmune diseases, such as SLE, have been also reported for MIP1β (178).

## Modulating the Innate Immune Response during Severe IAV Infection

**Figure 1:**
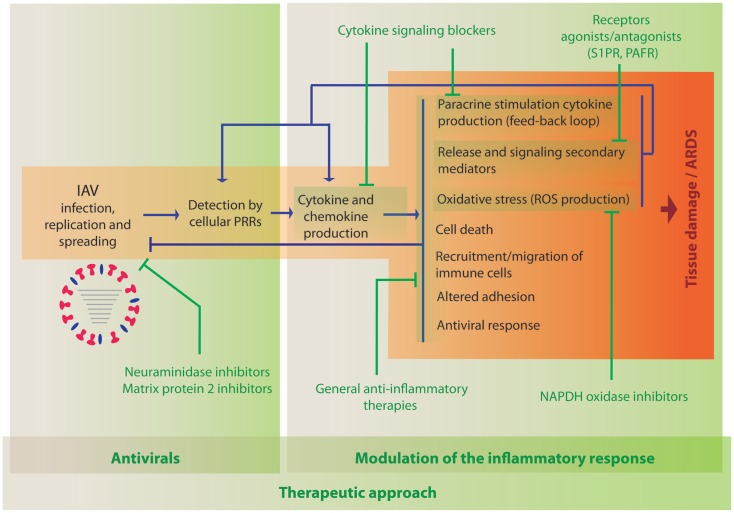
**Activation of innate immune processes by IAV and therapeutic opportunities to modulate the immune response**. When IAV invades a new host, it infects and replicates in cells of the respiratory tract. Cellular sensors, such as TLRs, RLRs, NLRs, and CLRs, recognize the virus PAMPs and initiate immune responses leading to the activation of defense mechanisms to counteract viral infection. The development of the inflammatory response is accompanied by multiple changes in gene expression that also result in damage of the infected tissue. Antiviral treatment is the first opportunity to reduce viral load and inflammation (indicated in green, left panel). The use of anti-inflammatory drugs to reduce cytokine- and chemokine-induced damage that could be used in combination with antiviral therapies is under investigation (indicated in green, right panel).

As described above, the current literature indicates a clear role for hypercytokinemia during severe IAV infection. Initially, cytokine production is induced following detection of the virus by cellular PRRs (Figure 1). Therefore, the primary treatment of IAV infection should be antiviral compounds, such as neuraminidase inhibitors, which will limit viral replication and spread, and therefore minimize the production of pro-inflammatory cytokines. Inflammation results in the induction of multiple cellular processes that lead to increased oxidative stress, apoptosis, necrosis, altered adhesion, and migration of immune cells to the lung. In addition, these processes lead to the release of additional secondary mediators and induction of cytokines, which results in amplified inflammation leading to increased damage (Figure 1). Therefore, it is worth considering therapies that modulate these detrimental processes in combination with antiviral agents. Targeting some of the most prevalent cytokines or related signaling pathways in severe influenza in mouse models, using either knock out animals or blocking agents, have been shown to reduce lung damage and mortality in multiple studies as indicated in the previous section, supporting the idea that anti-inflammatory agents inhibiting the same pathways could be beneficial in humans. One of the most important parameters that should be evaluated among these anti-inflammatory agents is that the treatment should reduce the negative effects of inflammation but not the innate and adaptive immune arms that are responsible for restricting viral replication and spread. However, the pathways initiated by the most prevalent cytokines are very redundant and dissecting these complex responses is very challenging. Specifically, blockade of TNFα and IL1β have shown a potential benefit in the mouse model, while blocking other cytokines, such as type I or III IFN or IL6-worsened disease outcome. Inhibition of specific chemokines or their receptors are also possible strategies. A few reports have been reported evaluating the consequence of blocking their function, which indicated that IP10 and MCP1 might have beneficial effects on reducing morbidity due to inflammation, while deficiency in RANTES expression seems to be detrimental. Further studies in animal models should be performed to better understand which of these pathways could be targeted as an anti-inflammatory therapy during severe influenza disease. In addition to cytokines and chemokines, other elements of the inflammatory response are under consideration for this purpose. In this section, we review those therapies that have been evaluated in the clinic or that have shown promising results in preclinical studies, such as broad-spectrum therapies, other signaling mediators or their receptors, or molecules involved in the generation of oxidative stress.

### Corticosteroids

Corticosteroid treatment has been proven to be safe in patients with ARDS and is associated with reduced inflammation and improved clinical status (179). For this reason, the use of these drugs has been considered for the treatment of severe influenza and has, in fact, been used in several cases of avian influenza (H5N1) virus infection (180). In addition, corticosteroids are regularly used in long-term treatment for asthma and chronic obstructive pulmonary disease (COPD). Thus, understanding the effect of these drugs during influenza virus infection is very relevant not only for their anti-inflammatory use in cases of IAV infection but also to determine the best methods to manage these high risk patients in the clinical setting.

Several studies have evaluated the consequences of using corticosteroids in humans with influenza infection, with varying results. A study by Quispe–Laime reported a reduction in lung injury and multiple organ dysfunctions in H1N1 influenza virus-infected patients treated with corticosteroids (181). However, as recently reviewed by Hui and Lee, several clinical trials indicate that the administration of these steroids during influenza virus infection, either in the presence or absence of neuraminidase inhibitors, has either no effect or even a detrimental effect (182). A retrospective study by Kudo and colleagues evaluated the effect of corticosteroid administration in patients with 2009 H1N1 IAV infection with pneumonia and did not find a negative effect of the steroid treatment (183). On the other hand, Lee et al., in a prospective study with adult patients infected with H3N2 IAV, showed that the administration of systemic corticosteroid to reduce exacerbation of asthma or in patients with COPD correlated with delayed viral clearance (184). Accordingly, another retrospective study in adults infected with 2009 H1N1 influenza virus showed an association of corticosteroid treatment with increased mortality (185). An interesting study by Thomas et al. showed that glucocorticosteroid treatment prior to IAV infection inhibits antiviral responses and the release of cytokines when tested in cultured primary human airway cells, which resulted in increased viral replication (186). Similarly, an *in vivo* experiment in the same study showed higher replication in a mouse model after treatment, which resulted in enhanced inflammation. This is in agreement with the recent meta-analysis of the literature performed by Zhang et al., where they concluded that corticosteroids are likely to increase mortality and morbidity by influenza 2009 H1N1 virus (187).

Consequently, the current literature suggests that the corticosteroid treatment is not a good choice for the treatment of acute inflammation during influenza virus infection, probably due to increased viral replication as a consequence of reduced antiviral responses (188). Accordingly, WHO discourage the use of corticosteroid drugs as routine treatment for severe influenza due to the paucity of evidence for beneficial effects (189). Further research in this field should be done, given the routine use of chronic corticosteroids treatment in some other medical conditions, such as asthma and COPD, both of which are considered high risk populations for influenza disease.

### Statins

Statins are also a class of drugs with extensive use in the clinic given their ability to decrease cholesterol levels, thus reducing the risk of cardiovascular disease. These drugs are inhibitors of the hydroxyl methylglutaryl-coenzyme A (HMG-CoA) reductase enzyme, acting in the cholesterol synthesis pathway. Interestingly, these drugs also have anti-inflammatory properties (190), which have been analyzed in the context of influenza infection (191). By altering the cholesterol synthesis route, statins also reduce the synthesis of lipid intermediates necessary for isoprenylation of multiple proteins. Consequently, multiple intracellular signaling pathways activated during the development of the inflammatory response are also affected (192).

An observational study that included more than 3000 patients hospitalized with influenza in the United States identified an association of statin use with reduced mortality (193). Other clinical studies have also shown that statin use could be beneficial in the treatment of influenza (194, 195), while yet other studies did not find supporting evidence for the use of this type of drug (196, 197). These retrospective studies, however, have the limitation that patients who are prescribed statins are some of those who are already at a higher risk for developing severe disease (due to pre-existing cardiovascular disease) and timing, duration and dose of the statin treatments are difficult to control. An interesting review by Mehrbod et al. (198) provides more detailed information on clinical trials evaluating the use of statins in IAV infections.

While the literature on this topic shows varied results, there is increasing evidence for a possible beneficial effect of the use of statins during influenza treatment, and further experimentation to confirm a positive effect should be developed. This is supported by several *in vitro* and *in vivo* studies that have indicated that, in addition to diminishing the production of cytokines upon influenza virus infection, statins also seem to result in decreased levels of viral replication (198–200).

### *N*-acetylcysteine

*N*-acetylcysteine is also a commonly used compound, which is mainly known for its mucolytic as well as anti-oxidant properties. Interestingly, anti-inflammatory properties have been also attributed to this molecule, which are probably associated with its anti-oxidant function by diminishing oxidative stress during inflammation. Related to this, animal models of systemic endotoxin-induced shock or acute lung injury showed reduced production of cytokines and tissue damage upon treatment with N-acetylcysteine (201–203).

Although the effect of this molecule in the context of influenza treatment has not been broadly studied, there are some reports indicating a possible beneficial effect. One study by Geiler et al. showed reduction of viral replication and pro-inflammatory cytokines in human lung epithelial cell lines upon infection with H5N1 influenza virus (204). The mechanism of inhibition seemed to be related to reduced NF-κB and MAPK p38 activation. These data were confirmed by a similar study where an H3N2 IAV and an influenza B virus strain were evaluated (205). Data from *in vivo* studies also seem to indicate that *N*-acetylcysteine might help to protect against IAV-induced pathology (206). It is important to note that in this case, contradictory reports have also been published, such as the study by Garigliany and Desmecht, which did not find an effect of the treatment in the mouse model (207). In humans, de Flora et al. (208) showed a long-term positive effect of *N*-acetylcysteine administration on the development of influenza or influenza-like symptoms. Therefore, although the amount of data reported is scarce, there seems to be evidence for a possible safe and beneficial effect for the use of *N*-acetylcysteine to treat inflammation by influenza without enhancing viral replication. However, studies evaluating this molecule in humans are very limited, and more extensive work is needed to obtain conclusive information.

### Macrolides

Macrolides, which are generally used for their antibacterial activity, also have immunomodulatory properties. They have been shown to reduce the expression of several cytokines and chemokines, such as IL6, IL8, and TNFα during different inflammatory processes. The ability of macrolides to interfere with multiple signaling pathways accounts for these immunomodulatory properties. For instance, some macrolides suppress NF-κB and AP-1 signaling (209, 210), affect intracellular Ca^2+^ dynamics (211), and inhibit the ERK1/2 pathway (212).

*In vitro* studies have shown that clarithromycin clearly reduces viral replication in epithelial cell lines approximately 4–7 h after viral adsorption (213). This effect is therefore also independent of the anti-inflammatory activity and might be mediated by alteration in cell signaling pathways. *In vivo* studies also support a potential role for the macrolides in improving recovery upon infection with IAV (214).

In the clinic, macrolides are sometimes administered in cases of influenza to treat secondary bacterial infections and because of their anti-inflammatory effects, clarithromycin being the most frequently prescribed first-line drug (215). Higashi et al. (216) analyzed the benefits of clarithromycin treatment in combination with neuraminidase inhibitors in patients with influenza infection. Their data indicated a possible effect in reducing fever, but they did not observe any differences in IL6 serum levels. However, another study could not find any association between significant improvement of symptoms and the use of macrolides.

In general, the number of studies evaluating macrolides in IAV infection is very limited. While *in vitro* and *in vivo* data showed promising results as indicated by a reduction of pro-inflammatory molecules alongside reduced viral replication, the small number of clinical studies does not suggest a significant benefit. Also, the use of antibiotics should be limited to cases with secondary bacterial infections, given the risk for emerging resistances. In addition, mice studies have shown that treatment with a combination of several antibiotics leads to impaired innate and adaptive immune responses and delayed virus clearance as a consequence of changes in the respiratory microbiota (217, 218) and therefore its use during influenza virus infection in humans should be further analyzed and cautiously used during severe infections.

### COX-2 inhibitors

Cyclooxygenase enzymes catalyze the conversion of arachidonic acid to prostaglandins, which play important roles in modulating immune responses and inflammation. While the isoform COX-1 is constitutively expressed, COX-2 is induced by several stimuli, such as LPS, pro-inflammatory cytokines, and growth factors (219). Importantly, COX enzymes are main targets for non-steroidal anti-inflammatory drugs including aspirin, ibuprofen, diclofenac, naproxen, and for selective COX-2 inhibitors, such as celecoxib and nimesulide, and are therefore very available and frequently used as treatment for other conditions.

Considering the well-described pro-inflammatory role of COX-2, studies to understand its function in influenza pathogenesis have been performed. COX-2 knock out mice infected with IAV showed reduced levels of pro-inflammatory cytokines and mortality, but also increased levels of replication (220). Interestingly, COX-1 ablation showed opposite results, with augmented and earlier inflammatory responses. COX-2 expression was observed to be elevated in autopsy tissue samples from patients infected by H5N1 IAV (182, 221). *In vitro* experiments have shown that COX-2 inhibitors play a regulatory role in mediating pro-inflammatory responses after H5N1 infection (221, 222) and have been shown to have a direct antiviral effect in human macrophages infected with H5N1 influenza virus (223). However, another *in vivo* study did not find a beneficial effect from celecoxib treatment in mice infected with an H3N2 virus. Therefore, data regarding COX-2 inhibitors are also controversial. Another *in vivo* study did observe a positive effect of celecoxib administration when used in combination with mesalazine or 5-aminosalicylic acid (another anti-inflammatory drug) in addition to a neuraminidase inhibitor in mice challenged with H5N1 IAV (224), supporting the idea that a combination treatment might be more efficient.

To date, there are no systematic human studies evaluating COX-2 inhibitors for influenza treatment. The event that these studies move forward is important to consider the selectivity for COX-2 inhibitors, since COX-1 inhibitors would have an opposite effect, increasing inflammation, and pathogenesis. Indeed, an increased risk of mortality during influenza virus infection was associated with aspirin, paracetamol, and diclofenac in animal models in a meta-analysis of the literature (225).

### Peroxisome proliferator-activated receptor agonists

Peroxisome proliferator-activated receptors (PPAR) are nuclear receptors and ligand-activated transcription factors that control a number of target genes upon assembly of a transcriptional complex. There are several PPAR, but in general, they are regulators of energy balance, including glucose homeostasis, fatty acid oxidation, and lipid metabolism, and are frequently used in the treatment of diabetes (226).

Several *in vivo* studies point to a possible benefit of the use of these drugs in treating influenza infection. Moseley at al. showed a reduction in morbidity and mortality in mice infected with two different H1N1 strains and treated with PPAR agonists (227). Similarly, PPAR agonist treatment of mice challenged with an H5N1 or an H2N2 IAV led to decreased inflammation and morbidity, and increased survival (228–230), using a cyclopentenone prostaglandin (prostanoid 15-deoxy-Δ12,14-prostaglandin-j2), observed a reduction in the levels of cytokines and chemokines in a mouse model of influenza in addition to a reduction in viral titers, and this effect was shown to be mediated by PPARγ (230).

While these drugs have not been thoroughly studied for influenza treatment and no human studies have been performed so far, exploring their potential would be of great interest given their current use in the clinic and availability, which would facilitate their study in clinical trials (231).

### Sphingosine-1-phosphate-1 receptor agonists

Sphingosine-1-phosphate (S1P) is a lipid signaling mediator synthesized from ceramides. The laboratory of Dr. Oldstone at The Scripps Research Institute (La Jolla, CA, USA) has focused on the use of S1PR agonists as a possible therapeutic to alleviate the inflammatory response arising during IAV infection, providing very interesting insights about the mechanisms of immunopathogenesis. They were first able to demonstrate that the administration of a promiscuous S1P receptor agonist led to a significant reduction of cytokines and chemokines upon influenza infection in the mouse model (232–234). This reduction of the inflammatory response correlated with a decrease in lung injury and improved survival upon infection (235). Importantly, the reduction of inflammation was not accompanied by a delayed clearance of the virus, indicating a potential for the use of these drugs as a therapeutic agent (234). Further work using S1PR agonists led them to describe a central role for endothelial cells in the generation of the cytokine storm (236). They further searched for the signaling pathways that the S1PR agonists might use to exert these anti-inflammatory-protective functions during IAV infection and found that the effect observed is independent of TLR3, TLR7, or cytosolic signaling pathways (237). In addition, they found an essential role for IL1R and MyD88/TRIF signaling in cytokine amplification (237). Therefore, although S1PR agonists are under investigation in mice and ferrets for influenza treatment (238), results from these studies are promising as a possible future treatment for hypercytokinemia in severe cases of influenza. One S1PR agonist has been approved in the clinic by the FDA for the treatment of relapsing–remitting multiple sclerosis. However, adverse effects have been noted in the use of this drug, and the safety profile of this and other S1PR agonists should be further investigated (239).

### Platelet-activating factor receptor antagonists

Platelet-Activating Factor (PAF) is a phospholipid mediator involved in many cellular processes including cell motility and synthesis of cytokines and other signaling mediators (240). PAF signaling occurs through the PAF receptor (PAFR), which is a single GPCR, expressed in the plasma and nuclear membranes of leukocytes, endothelial cells, epithelial cells, smooth muscle cells, and platelets (240). It is known that expression of PAFR in the airway is upregulated by IAV infection, and it is believed that this facilitates bacterial adherence and therefore susceptibility to *Streptococcus pneumonia* (241).

The use of PAFR antagonists has been proposed in different pathological settings, including influenza, mainly due to their anti-inflammatory properties (242). Using *PAFR* knock out mice and antagonists, Garcia et al. demonstrated that eliminating or counteracting these receptors reduced lung injury, infiltration of mononuclear cells and neutrophils, and the expression of IL12, RANTES, and IFNγ while not affecting the levels of IL6 and increasing IL1β production (243). This overall reduced immune response did not result in an elevated level of viral replication. A mechanistic analysis showed activation of TLR7/8 during infection was dependent on PAFR. While according to these data, PAFR antagonists could be candidates to treat inflammation during influenza, further characterization of the effect of these drugs should be performed.

### Other candidates

Other anti-inflammatory therapies have been tested in animal models resulting in reduced inflammation, morbidity, and mortality. While these studies support the potential positive effect of immunomodulatory therapy in severe influenza, the scientific data in this field are very preliminary, and extensive investigation is needed to develop these treatments for human use. Here we discuss some of these treatments.

NADPH oxidases, enzymes that are involved in ROS production, have also been proposed as targets for reducing IAV-induced inflammation. There is evidence that activation of NOX2 promotes lung oxidative stress, inflammation, injury, and dysfunction resulting from infection with IAV ranging from low to high pathogenicities (244). Apocynin, a NOX2 inhibitor, inhibited influenza-induced hypercytokinemia and ROS production in airway epithelial and immune cells *in vitro*, while not affecting viral replication (41).

A study by Sharma et al. analyzed the effect of other two orally available and approved anti-inflammatory drugs, a phosphodisesterase-4 inhibitor and a selective serotonin reuptake inhibitor. This study showed a clear reduction in the levels of cytokines and chemokines, lung infiltration, alveolitis, and overall lower mortality in H1N1-infected mice, all while not affecting the levels of viral replication (245).

Another research group further explored the combination of antiviral and anti-inflammatory therapy and generated a novel compound with these two properties by conjugating two drugs, zanamivir (a neuraminidase inhibitor) and caffeic acid (cytokine suppressor) (246). This innovative method provided improved protection in mice against H1N1 and H5N1 IAV.

## Concluding Remarks

There is substantial information in the literature supporting the association of influenza pathogenesis with high levels of inflammation and production of cytokines and chemokines, highlighting the opportunity to identify immunomodulatory drugs that could reduce the inflammation-associated damage in the lung seen in severe cases of influenza. These therapies should be evaluated in combination with antivirals, which control virus replication and spread. Reduction of viral load with antiviral drugs also acts to decrease inflammation by lowering the presence of PAMPs to be sensed by cellular PRRs. In addition, one crucial aspect to assess when testing these drugs is to assure that the treatment does not provide an environment for enhanced replication due to a general shutdown of the innate and adaptive immunity.

To date, the therapies studied in humans have commonly used broad-spectrum anti-inflammatory drugs, which are frequently used for other affections. Corticosteroids are a good example of those therapies, which are frequently used in patients with asthma and COPD, and have been evaluated in multiple studies with conflicting results. Some of those studies point to a possible detrimental role of treatment with corticosteroids, and their use should be avoided if possible until their effect is better understood. Other broad-spectrum anti-inflammatory drugs that could be beneficial are statins, *N*-acetylcysteine, and COX-2 inhibitors. However, there is no sufficient data in the current literature to justify their use. More specific treatments that have been explored in animal models include blocking cytokines, such as TNFα or IL1β, reducing the oxidative stress through NADPH inhibitors, or the use of inhibitors for receptors for secondary inflammatory mediators, such as PAFR or S1PR. As for the last examples, targeting cell surface receptors in immune cells is an attractive approach since this would facilitate cellular accessibility of the drug. Further research to bring these therapies closer to the clinic in the context of IAV infection is needed, as well as for the identification of novel immunomodulatory agents.

## Conflict of Interest Statement

The authors declare that the research was conducted in the absence of any commercial or financial relationships that could be construed as a potential conflict of interest.
